# Supplementary figures and images for: Biased exonization of transposed elements in duplicated genes: A lesson from the TIF-IA gene
Source: BMC Mol Biol. 2007 Nov 29;8:109. doi: 10.1186/1471-2199-8-109 (PMC2231382; doi:10.1186/1471-2199-8-109)

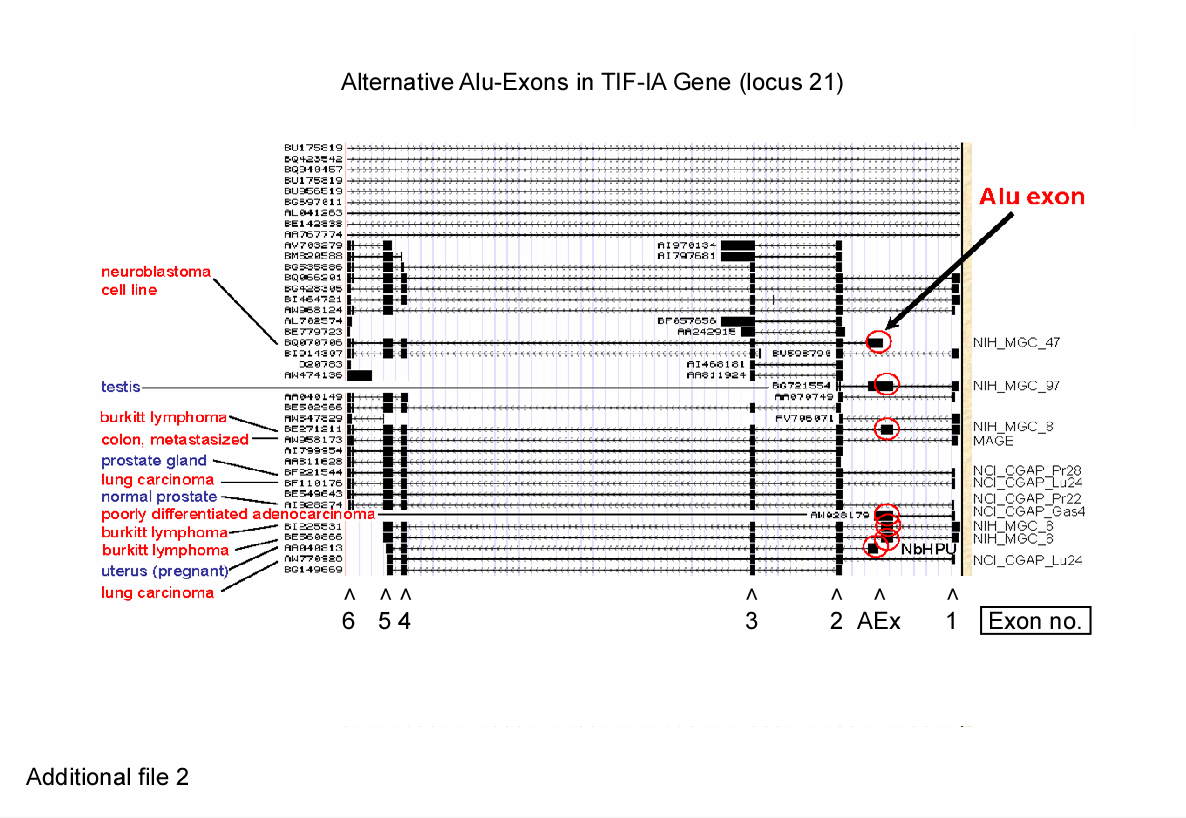

Supplement: Additional file 2 — Indications of exonization only from locus 21. Alignment of EST/cDNA with the human genome using UCSC Genome Browser reveals alternative L2-AExs between exon 1 and 2. The L2-AExs' are marked with red circles. Exons' numbers are indicated on the bottom. Seven out of the 19 ESTs that cover that locus contain an L2-AEx, of which 5 were originated from cancerous sources (3 from Burkitt's lymphoma, 1 from neuroblastoma cell line, and 1 from poorly differentiated adenocarcenoma). The rest of the Alu-containing spliced isoforms originated from reproductive organs (testis and uterus). The alignment indicates that the L2-AEx is also alternatively 5' spliced. [file 1471-2199-8-109-S2.tiff]

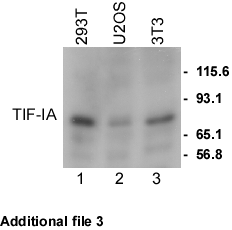

Supplement: Additional file 3 — The WT TIF-IA protein is synthesized in cells with and without the exonization. Total protein was extracted from the indicated cells and analyzed by Western blotting using polyclonal rabbit anti-TIF-IA serum (kindly gifted from the Grummt's lab) at a 1/10,000 dilution. 293T and U2OS are human cell lines, and 3T3 is a murine cell line. [file 1471-2199-8-109-S3.tiff]
